# Photonic helicoid-like surface states in chiral metamaterials

**DOI:** 10.1038/s41598-023-40926-8

**Published:** 2023-08-25

**Authors:** Ruey-Lin Chern

**Affiliations:** Institute of Applied Mechanics, National Taiwan University, Taipei, 106 Taiwan

**Keywords:** Nanophotonics and plasmonics, Metamaterials

## Abstract

We investigate the photonic topological phases in chiral metamaterials characterized by the magnetoelectric tensors with diagonal chirality components. The underlying medium is considered a photonic analogue of the topological semimetal featured with a Weyl cone and a cylindrical surface in the frequency-wave vector space. As the ’spin’-degenerate condition is satisfied, the photonic system can be rearranged as two hybrid modes that are completely decoupled. By introducing the pseudospin states as the basis for the hybrid modes, the photonic system is described by two subsystems in the form of spin-orbit Hamiltonians of spin 1, which result in nonzero spin Chern numbers that determine the topological properties. Surface modes at the interface between vacuum and the chiral metamaterial exist in their common gap in the wave vector space, which are analytically formulated by algebraic equations. In particular, the surface modes form a pair of spiral surface sheets wrapping around the Weyl cone, resembling the helicoid surface states that occur in topological semimetals. At the Weyl frequency, the surface modes contain two Fermi arc-like states that concatenate to yield a straight line segment.

## Introduction

Topological phases are new phases of matter characterized by integer quantities known as topological invariants, which remain constant under arbitrary continuous deformations of the system. Quantum Hall (QH) state^1^ is the very first example of two dimensional (2D) topological phase, belonging to the class with broken time-reversal (TR) symmetry because of the presence of a static magnetic field. Quantum spin Hall (QSH) state^2–4^ is a different 2D topological phase without the magnetic field and preserves the TR symmetry, where the spin-orbit coupling is responsible for the topological characters. Topological properties of the QH states are characterized by TKNN invariants or Chern numbers^5^, while those of the QSH states are characterized by $Z_2$ invariants^2^ or spin Chern numbers^6^. Theoretical concepts developed in the QSH states are generalized to three dimensions (3D), leading to the more general class of 3D topological insulators^7,8^.

One remarkable feature of the QSH state is the emergence of gapless edge states inside the bulk band gap. The propagation direction of the edge states is locked by the spin^9^, which enables topologically protected edge states that propagate unidirectionally without back scattering^10^. As the edge states are protected by the bulk topology, they are insensitive to small perturbations that do not change the topology. Similar to the case of 2D topological phases, gapless surface states appear inside the band gap between two topologically distinct bands in 3D topological insulators^11,12^, which can be realized in both TR broken^13,14^ and TR invariant systems^15–17^. In contrast to 3D topological insulators that are gapped topological phases, 3D *gapless* topological phases are a new type of phases known as topological semimetals^18–22^.

A majority of the topological semimetals are characterized by Weyl degeneracies, which are degeneracies between topologically inequivalent bands. The main signature of 3D gapless topological phases is the appearance of Weyl points existing in the systems that lack TR symmetry, inversion symmetry, or both. The Weyl points are understood as the monopoles of Berry curvature in the momentum space that carry quantized topological charges, which are equal to the topological invariants of the system. A useful perspective on the Weyl semimetals is to view them as the transitional state between a topological insulator and a trivial insulator^22^. An important feature of the Weyl points is the existence of Fermi arcs that connect the Weyl points, corresponding to the topologically protected surface states that are robust against disorder. In particular, surface states may form a spiral surface sheet that connects the upper and lower bulk cones, which are protected from being gapped by nonsymmorphic symmetries and termed as *helicoid* surface states^23^.

The novel concepts of topological phases have been extended to photonic systems^24–26^, leading to the discovery of photonic QH states^27–31^, photonic QSH states^32–36^, photonic topological insulators^37–39^ and photonic topological semimetals^40–47^. The key aspect to construct a topological phase is having a Kramers pair in the system, which are doubly degenerate eigenstates under TR symmetry^48^. The Kramers theorem, however, is usually valid for a TR invariant system of spin 1/2^10^ and cannot readily apply to the photonic system of spin 1^49,50^, unless additional symmetry has been imposed. Nevertheless, photons have spin properties as a result of circular polarization^51^. A spin-like quantity called *pseudospin* can be formed by the linear combination of electric and magnetic fields when a specific degenerate condition between the electric and magnetic parameters is satisfied^32^. As a result, the photonic system can be described by an effective Hamiltonian consisting of two subsystems for the pseudospin states^32–34^, and the Kramers pair can be formed in the photonic system. In the presence of chirality or bianisotropy that emulates the spin-orbit coupling, a topological phase can be constructed in the photonic system^52–56^.

In the present study, we investigate the photonic topological phases in chiral metamaterials characterized by the magnetoelectric tensors with diagonal chirality components. Bulk modes of the underlying medium are represented by two decoupled quadratic equations when a certain symmetry of the material parameters is included. As the ‘spin’-degenerate condition^32,34,38^ is satisfied, the bulk modes are featured with a Weyl cone and a cylindrical surface in the frequency-wave vector space. The electromagnetic duality allows for Maxwell’s equations to be decoupled into two subsystems for the *hybrid* modes, which are defined as the linear combinations of electric and magnetic fields. By introducing the pseudospin states as the basis for the hybrid modes, the photonic system can be described by a pair of spin-orbit Hamiltonians of spin 1^53–59^ that respect the fermionic-like *pseudo* TR symmetry. The topological properties of the photonic system are determined by nonzero spin Chern numbers calculated from the eigenfields of the Hamiltonians. Surface modes at the interface between vacuum and the chiral metamaterial exist in their common gap in the wave vector space, which are analytically formulated by algebraic equations. In particular, the surface modes are represented by a pair of spiral surface sheets wrapping around the Weyl cone, resembling the helicoid-like surface states that occur in the topological semimetals^23^. At the Weyl frequency, the surface modes contain two Fermi arc-like states that concatenate to yield a straight line segment.

## Results

### Bulk modes

Consider a general bianisotropic medium characterized by the constitutive relations:

1 $$\begin{aligned} {\textbf{D}}&= \varepsilon _0\underline{\varepsilon } {\textbf{E}} + \sqrt{{\varepsilon _0}{\mu _0}} \underline{\xi } {\textbf{H}}, \end{aligned}$$

2 $$\begin{aligned} {\textbf{B}}&= \mu _0\underline{\mu } {\textbf{H}} + \sqrt{{\varepsilon _0}{\mu _0}} \underline{\zeta } {\textbf{E}}, \end{aligned}$$

where $\underline{\varepsilon }$, $\underline{\mu }$, $\underline{\xi }$ and $\underline{\zeta }$ are the frequency-dependent relative permittivity, permeability, and magnetoelectric tensors, respectively. Treating the combined electric field $\textbf{E}=\left( E_x,E_y,E_z\right) ^T$ and magnetic field $\textbf{H}=\left( H_x,H_y,H_z\right) ^T$ as a six-component vector, where *T* denotes the transpose, Maxwell’s equations for the time-harmonic electromagnetic fields (with the time convention ${e^{-i\omega t} }$) are written in matrix form as

3 $$\begin{aligned} \left( {\begin{array}{*{20}{c}} {{\omega }\underline{\varepsilon }} &{} {c\mathbf{{k}} \times \underline{I} + {{\omega }\underline{\xi }}} \\ {-c\mathbf{{k}} \times \underline{I} + {{\omega }\underline{\zeta }}} &{} { {\omega }\underline{\mu }} \\ \end{array}} \right) \left( {\begin{array}{*{20}{c}} \mathbf{{E}} \\ \mathbf{{H}'} \\ \end{array}} \right) = 0, \end{aligned}$$

where $\underline{I}$ is the 3 $\times$ 3 identity matrix and $\textbf{H}'=\eta _0\textbf{H}$, with ${\eta _0} = \sqrt{{\mu _0}/{\varepsilon _0}}$. Let the medium be lossless ($\underline{\varepsilon }=\underline{\varepsilon }^\dagger$, $\underline{\mu }=\underline{\mu }^\dagger$, and $\underline{\xi }=\underline{\zeta }^\dagger$, where $\dagger$ denotes the Hermitian conjugate) and reciprocal ($\underline{\varepsilon }=\underline{\varepsilon }^T$, $\underline{\mu }=\underline{\mu }^T$, and $\underline{\xi }=-\underline{\zeta }^T$, where *T* denotes the transpose)^60^, which implies that $\underline{\varepsilon }=\underline{\varepsilon }^*$, $\underline{\mu }=\underline{\mu }^*$, $\underline{\xi }=-\underline{\xi }^*$, and $\underline{\zeta }=-\underline{\zeta }^*$, where $*$ denotes the complex conjugate. In the present study, we further assume that the permittivity, permeability, and magnetoelectric tensors are uniaxial: $\underline{\varepsilon }=\mathrm{{diag}}\left( {{\varepsilon _t},{\varepsilon _t},{\varepsilon _z}}\right)$, $\underline{\mu }=\mathrm{{diag}}\left( {{\mu _t},{\mu _t},{\mu _z}}\right)$, and $\underline{\xi }=- {\underline{\zeta }}=\mathrm{{diag}}\left( {i{\gamma _t},i{\gamma _t},i{\gamma _z}}\right)$, where $\varepsilon _n$, $\mu _n$, and $\gamma _n$ $(n=t,z)$ are real-valued quantities, and $\textrm{diag}\left( \varvec{\cdot },\varvec{\cdot },\varvec{\cdot }\right)$ denotes a 3 $\times$ 3 diagonal matrix. The medium with the purely imaginary magnetoelectric tensors is known as the *chiral* medium. Here, the chirality parameter $\gamma _n$ ($n=t,z$) appears in the diagonal elements of the magnetoelectric tensors $\underline{\xi }$ and $\underline{\zeta }$, which means that the magnetoelectric couplings of the chiral medium occur in parallel directions. The underlying medium can be synthesized by metallic helices oriented along three perpendicular directions^61^. In the chiral medium, the inversion symmetry is broken because of the chirality^44,62^, whereas the TR symmetry is preserved^24^.

The existence of a nontrivial solution of $\textbf{E}$ and $\textbf{H}$ requires that the determinant of the 6 $\times$ 6 matrix in Eq. (3) be zero, giving rise to the characteristic equation of bulk modes as

4 $$\begin{aligned} \begin{aligned}{}&\left( {{\varepsilon _t}{\mu _t} - \gamma _t^2} \right) \left( {k_x^4 + k_y^4} \right) + \left( {{\varepsilon _z}{\mu _z} - \gamma _z^2} \right) k_z^4 \\&+ \left( {{\varepsilon _z}{\mu _t} + {\varepsilon _t}{\mu _z} - 2{\gamma _t}{\gamma _z}} \right) \left( {k_x^2 + k_y^2} \right) k_z^2 + 2\left( {{\varepsilon _t}{\mu _t} - \gamma _t^2} \right) k_x^2k_y^2 \\&- \left( {{\varepsilon _t}{\mu _t} - \gamma _t^2} \right) \left( {{\varepsilon _z}{\mu _t} + {\varepsilon _t}{\mu _z} + 2{\gamma _t}{\gamma _z}} \right) \left( {k_x^2 + k_y^2} \right) k_0^2 \\&- 2\left( {{\varepsilon _t}{\mu _t} + \gamma _t^2} \right) \left( {{\varepsilon _z}{\mu _z} - \gamma _z^2} \right) k_z^2k_0^2 + {\left( {{\varepsilon _t}{\mu _t} - \gamma _t^2} \right) ^2}\left( {{\varepsilon _z}{\mu _z} - \gamma _z^2} \right) k_0^4 = 0, \end{aligned} \end{aligned}$$

where ${k_0} = \omega /c$. This is a bi-quadratic equation that incorporates the coupling between transverse electric and transverse magnetic modes. If $\eta _t=\eta _z$, that is, $\sqrt{\mu _t/\varepsilon _t}=\sqrt{\mu _z/\varepsilon _z}$, Eq. (4) can be decoupled as a product of two quadratic equations as

5 $$\begin{aligned} \left( {\frac{{k_t^2}}{{n_t^ + n_z^ + }} + \frac{{k_z^2}}{{{{\left( {n_t^ + } \right) }^2}}} - k_0^2} \right) \left( {\frac{{k_t^2}}{{n_t^ - n_z^ - }} + \frac{{k_z^2}}{{{{\left( {n_t^ - } \right) }^2}}} - k_0^2} \right) = 0, \end{aligned}$$

where $k_t^2 = k_x^2 + k_y^2$, $n_t^ \pm = \sqrt{{\varepsilon _t}{\mu _t}} \pm |{\gamma _t}|$, and $n_z^ \pm = \sqrt{{\varepsilon _z}{\mu _z}} \pm |{\gamma _z}|$. In the isotropic case, where $\varepsilon _t=\varepsilon _z\equiv \varepsilon$, $\mu _t=\mu _z\equiv \mu$, and $\gamma _t=\gamma _z\equiv \gamma$, Eq. (5) can be simplified to

6 $$\begin{aligned} \left( {{k_t^2+k_z^2} - n_ + ^2k_0^2} \right) \left( {{k_t^2+k_z^2} - n_ - ^2k_0^2} \right) = 0, \end{aligned}$$

where ${n_ \pm } = \sqrt{\varepsilon \mu } \pm |\gamma |$. There exists a *critical* condition: $|\gamma |=\sqrt{\varepsilon \mu }$, where $n_-=0$ and the corresponding bulk mode is reduced to a point. In case $\gamma =0$, Eq. (6) is further simplified to

7 $$\begin{aligned} \left( {{k_t^2+k_z^2} - \varepsilon \mu k_0^2} \right) ^2= 0. \end{aligned}$$

Note that the features of bulk modes may change with the frequency for a dispersive medium (usually the case of metamaterials), depending on the choice of frequency range. In the neighborhood of a *reference* frequency $\omega _\text {ref}$, ${\varepsilon _n}$ ($n=t,z$) can be approximated as ${\varepsilon _n} \approx {\varepsilon _{n0}} + {\left. {\frac{{d{\varepsilon _n}}}{{d\omega }}} \right| _{\omega = {\omega _\text {ref}}}}\left( {\omega - {\omega _\text {ref}}} \right) \equiv {\varepsilon _{n0}} + {\tilde{\varepsilon }_n}\delta \omega /{\omega _\text {ref}}$, where ${\tilde{\varepsilon }}_n$ is positive definite^57^. A similar relation is valid for $\mu _n$ ($n=t,z$). We further assume that the chirality parameter $\gamma$ varies smoothly around $\omega _{\textrm{ref}}$ and can be treated as a constant in the analysis^32,53,58^.

*Spin-orbit Hamiltonians* The electromagnetic duality of Maxwell’s equations dictates that the matrix in Eq. (3) holds a symmetric pattern when the ’spin’-degenerate condition $\underline{\varepsilon }=\underline{\mu }$^32,34,38^ is satisfied. This allows us to rewrite Eq. (3) as

8 $$\begin{aligned} \left( {\begin{array}{*{20}{c}} {{{ \mathscr{H}_0^+}}} &{} \mathbf{{0}} \\ \mathbf{{0}} &{} {{{ \mathscr{H}_0^-}}} \\ \end{array}} \right) \left( {\begin{array}{*{20}{c}} {\mathbf{{F}^+} } \\ {\mathbf{{F}^-} } \\ \end{array}} \right) = 0, \end{aligned}$$

where ${{\mathscr{H}_0^\pm }} = \mp {\omega }\underline{\varepsilon } + i \left( {c\textbf{k}} \times \underline{I}+\omega \underline{\xi }\right)$ and $\textbf{F}^\pm ={\mathbf{{E}} \pm i \mathbf{{H'}}}$ are the *hybrid* modes that linearly combine the electric and magnetic fields. Note that $\textbf{F}^+$ and $\textbf{F}^-$ are completely decoupled and determined by two subsystems ($3\times 3$ matrices) with a similar form. By introducing the *pseudospin* states ${\psi _ \pm } = {U^{ - 1}}{\tilde{\psi }_ \pm }$ as the basis for the hybrid modes, where $\tilde{\psi _ \pm } = {\left( - {\frac{{ {F_x^\pm } \mp i{F_y^\pm }}}{{\sqrt{2} }},{F_z},\frac{{{F_x^\pm } \pm i{F_y^\pm }}}{{\sqrt{2} }}} \right) ^T}$ and $U = \mathrm{{diag}}\left( {\sqrt{{{\tilde{\varepsilon }}_z}/{{\tilde{\varepsilon }}_t}} ,1,\sqrt{{{\tilde{\varepsilon }}_z}/{{\tilde{\varepsilon }}_t}} } \right)$, Eq. (8) can be formulated as a pair of eigensystems when the frequency dispersion of the medium near the reference frequency $\omega _\text {ref}$ is taken into account. In the isotropic case, where ${\varepsilon _{t0}} = {\varepsilon _{z0}} \equiv \varepsilon$ and ${\tilde{\varepsilon }_t} = {\tilde{\varepsilon }_z} \equiv \tilde{\varepsilon }$, the eigensystems for Eq. (8) are given by (see Methods A)

9 $$\begin{aligned} {\mathscr {H}_ \pm }{\psi _ \pm } - \mathscr{D}_\pm {\psi _ \pm } = \pm \delta \omega {\psi _ \pm }, \end{aligned}$$

where

10 $$\begin{aligned} \mathscr{{H}_ +} = v \textbf{{k}} \cdot \textbf{{S}},\quad \mathscr{{H}_ -} = - v {\textbf{{k}} } \cdot {\textbf{{S}}^*}, \end{aligned}$$

and $\mathscr{D}_\pm = \pm {\omega _{\textrm{ref}}} \left( {\varepsilon \pm \gamma /\tilde{\varepsilon }}\right)$. Here, $v=c/{{\tilde{\varepsilon }}}$, $\mathbf{{k}}=k_x\hat{x}+k_y\hat{y}+k_z\hat{z}$, $\mathbf{{S}} = {S_x}\hat{x} + {S_y}\hat{y} + {S_z}\hat{z}$, with $S_n$ ($n=x,y,z$) being the spin matrices of spin 1. Note that Eq. (9) is expressed as an eigensystem with $\pm \delta \omega$ being the eigenvalue. The Hamiltonian $\mathscr{H}_\pm$ in Eq. (10) represents the spin-orbit coupling $\mathbf{{k}}\cdot \mathbf{{S}}$ of spin 1, which is mathematically equivalent to the Hamiltonian of a magnetic dipole moment in the magnetic field^57^.

*Topological invariants* The topological properties of the spin-orbit Hamiltonians $\mathscr{H}_\pm$ can be characterized by the topological invariants based on the eigenfields. For this purpose, we calculate the Berry flux over a closed surface in the wave vector space. The eigensystem for the Hamiltonian $\mathscr{H}_\pm$ in Eq. (10):

11 $$ \begin{aligned} {\mathscr {H}_ \pm }\psi _ \pm ^\sigma =\lambda _ \pm ^\sigma \psi _ \pm ^\sigma \end{aligned}$$

is solved to give the eigenvalues $\lambda _ \pm ^\sigma$ and eigenvectors $\psi _ \pm ^\sigma$ ($\sigma =\pm 1, 0$). Here, the eigenvalue $\lambda _ \pm ^\sigma$ is related to $\delta \omega$ in Eq. (9) as $\lambda _ \pm ^\sigma = \mathscr{D}_\pm \pm \delta \omega$. Based on Eq. (11), the Chern numbers are calculated to give (see Methods B)

12 $$\begin{aligned} {C_\sigma } = 2\sigma . \end{aligned}$$

The nonzero $C_\sigma$ ($\sigma =\pm 1$) characterizes the topological properties of the system, where $\sigma$ refers to the helicity (or handedness) of the pseudospin states. In particular, the edge or surface states at the interface between two distinct topological phases are topologically protected, which means that their existence is guaranteed by the difference in band topology on two sides of the interface. In this system, the total Chern number $C=\sum \limits _\sigma {{C_\sigma }}=0$ and the spin Chern number $C_{\textrm{spin}}=\sum \limits _\sigma {{\sigma C_\sigma }}=4$, which are consistent with the quantum spin Hall effect of light^51^. The topological invariants remain constant under arbitrary continuous deformations of the system. The topological properties in the isotropic case will be retained when a certain anisotropy is included in the system. For a more general anisotropic case, the exact calculation of topological invariants can be obtained by numerical integration of the Berry curvatures^63^.

### Pseudo time-reversal symmetry

The Hamiltonian for Maxwell’s equations [cf. Eq. (3)] in the chiral medium, which is lossless and reciprocal, is TR invariant under $T_b$, that is,

13 $$\begin{aligned} \left( {T_b \otimes \underline{I}}\right) {\mathscr{H}_m \left( \textbf{k} \right) }\left( {T_b \otimes \underline{I}}\right) ^{ - 1} = {\mathscr{H}_m\left( -\textbf{k} \right) }, \end{aligned}$$

where

14 $$\begin{aligned} {\mathscr{H}_m \left( \textbf{k} \right) } = \left( {\begin{array}{*{20}{c}} {{\omega }\underline{\varepsilon }} &{} {c\mathbf{{k}} \times \underline{I} + {{\omega }\underline{\xi }}} \\ {-c\mathbf{{k}} \times \underline{I} + {{\omega }\underline{\zeta }}} &{} { {\omega }\underline{\mu }} \\ \end{array}} \right) , \end{aligned}$$

${T_b} = {\sigma _z}K$ (with $T_b^2=1$) is the bosonic TR operator for photons^24^, with *K* being the complex conjugation, and $\otimes$ denotes the tensor product. The Hamiltonian $\mathscr{H}_m$, however, is not TR invariant under $T_f$, that is, $\left( {T_f \otimes I}\right) {\mathscr{H}_m \left( \textbf{k} \right) }\left( {T_f \otimes I}\right) ^{ - 1} \ne {\mathscr{H}_m\left( -\textbf{k} \right) }$, where ${T_f} = {i\sigma _y}K$ (with $T_f^2=-1$) is the fermionic TR operator for electrons^24^. Nevertheless, the combined Hamiltonian formed by two spin-orbit Hamiltonians $\mathscr H_\pm$ [cf. Eq. (10)] is TR invariant under $T_p$, that is,

15 $$\begin{aligned} \left( {T_p \otimes \underline{I}}\right) {\mathscr{H}_c \left( \textbf{k} \right) }\left( {T_p \otimes \underline{I}}\right) ^{ - 1} = {\mathscr{H}_c \left( -\textbf{k} \right) }, \end{aligned}$$

where

16 $$\begin{aligned} {{\mathscr {H}}_c}\left( {\textbf{k}} \right) = \left( {\begin{array}{*{20}{c}} {v {{\textbf{k}}\cdot {\textbf{S}}} } &{} {\textbf{0}} \\ {\textbf{0}} &{} { - v {{ {{\textbf{k}}\cdot {\textbf{S}}} }^*}} \\ \end{array} } \right) , \end{aligned}$$

and ${T_p}$ is the fermionic-like *pseudo* TR operator having the same form of $T_f$. The pseudo TR operator $T_p$ is inspired by noticing that $\mathbf{{E}} \leftrightarrow \mathbf{{H}}$ during the TR operation, which is defined as ${T_p} = {T_b}{\sigma _x} = {\sigma _z}K{\sigma _x} = i{\sigma _y}K$ with $T_p^2=-1$^34^. Here, $\sigma _x=\left( 0,1;1,0\right)$, $\sigma _y=\left( 0,-i;i,0\right)$, and $\sigma _z=\textrm{diag}\left( 1,-1\right)$ are the Pauli matrices. The pseudo TR symmetry of the combined Hamiltonian $\mathscr{H}_c$ is crucial in constructing the Kramers pair and determining the topological phases in photonic systems of spin 1, allowing for the existence of spin-polarized counterpropagating helical edge states as in electronic systems.

### Surface modes

Topological phase transition occurs between two distinct topological phases. In the present study, the topological phase transition is manifest on the existence of surface modes at the interface between vacuum and the chiral metamaterial. Here, we begin with the linear combination of eigenfields based on Maxwell’s equations, leaving the weighting coefficients as unknown variables. Applying the boundary condition at the interface, a condition can be arrived by requiring these coefficients to be nontrivial, which gives rise to the characteristic equation of surface modes.

Let the *xz* plane be an interface between vacuum ($y>0$) and the chiral medium ($y<0$) characterized by $\varepsilon _n=\varepsilon$, $\mu _n=\mu$, and $\gamma _n=\gamma$ ($n=t,z$), where the surface modes may exist. According to Maxwell’s boundary conditions: the continuity of tangential electric and magnetic field components at the interface, the characteristic equation of surface modes can be analytically formulated by using the eigenfields of bulk modes on two sides of the interface, given by (see Methods C)

17 $$\begin{aligned} \begin{aligned}{}&2\sqrt{\varepsilon }\sqrt{\mu }{k_x}{k_z}{k_0}\left[ {\sqrt{\varepsilon }\sqrt{\mu }\left( {\varepsilon \mu - 1} \right) \left( {k_y^{(1)} - k_y^{(2)}} \right) - {\gamma ^2}\sqrt{\varepsilon }\sqrt{\mu }\left( {k_y^{(1)} - k_y^{(2)}} \right) } \right. \\&\left. { + {\gamma ^3}\left( {k_y^{(1)} + k_y^{(2)}} \right) + \gamma \left( {2\left( {\varepsilon + \mu } \right) k_y^{(0)} - \left( {\varepsilon \mu + 1} \right) } \right) \left( {k_y^{(1)} + k_y^{(2)}} \right) } \right] \\&+ ik_0^2\left[ {2\sqrt{\varepsilon }\sqrt{\mu }n _ + ^2n _ - ^2k_x^2 + \left( {\varepsilon + \mu } \right) {n _ + }{n _ - }k_y^{(0)}\left( {{n _ - }k_y^{(1)} + {n _ + }k_y^{(2)}} \right) } \right. \\&+ \left. {2\sqrt{\varepsilon }\sqrt{\mu }\left( {2\left( {\varepsilon \mu + {\gamma ^2}} \right) k_z^2 - {n _ + }{n _ - }k_y^{(1)}k_y^{(2)}} \right) } \right] - 2i\sqrt{\varepsilon }\sqrt{\mu }n _ + ^2n _ - ^2k_0^4 \\&- ik_z^2\left( {2\sqrt{\varepsilon }\sqrt{\mu }k_x^2 + \left( {\varepsilon + \mu } \right) k_y^{(0)}\left( {{n _ + }k_y^{(1)} + {n _ - }k_y^{(2)}} \right) + 2\sqrt{\varepsilon }\sqrt{\mu }\left( {k_z^2 - {n _ + }{n _ - }k_y^{(1)}k_y^{(2)}} \right) } \right) = 0, \end{aligned} \end{aligned}$$

where ${n _ \pm } = \sqrt{\varepsilon \mu } \pm \gamma$, $k_y^{(0)} = \sqrt{k_0^2 - k_x^2 - k_z^2}$ is the normal wave vector component (to the interface) in vacuum, $k_y^{(1)} = - \sqrt{n _ + ^2k_0^2 - k_x^2 - k_z^2}$ and $k_y^{(2)} = - \sqrt{n _ - ^2k_0^2 - k_x^2 - k_z^2}$ are the normal wave vector components in the chiral medium, and the superscripts (1) and (2) refer to two independent polarizations.

**Figure 1:**
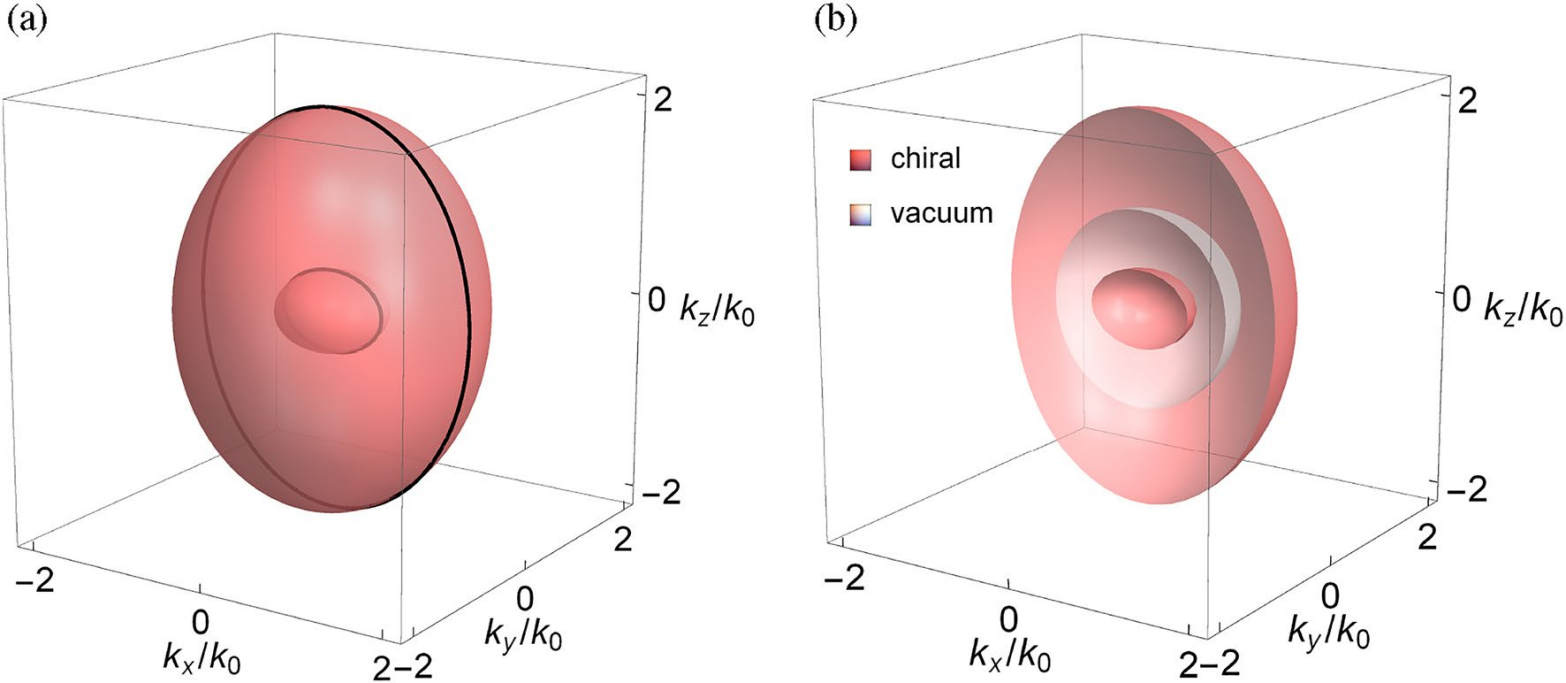
Equifrequency surfaces of the bulk modes in the wave vector space for the chiral metamaterial with (**a**) $\varepsilon _t=\mu _t=1.2$, $\varepsilon _z=\mu _z=1$, $\gamma _t=\pm 0.8$, and $\gamma _z=\pm 0.2$ (**b**) $\varepsilon _t=\mu _t=0.8$, $\varepsilon _z=\mu _z=0.2$, $\gamma _t=\pm 1.2$, and $\gamma _z=\pm 1$. In (**a**), black contours are bulk modes at $k_y=0$. In (**b**), bulk modes are shown for the half space with $k_y>0$.

## Discussion

### Bulk modes

Figure 1a shows the equifrequency surfaces of the bulk modes for the chiral metamaterial in the wave vector space based on Eq. (5). In the present study, we assume that $n_t^\pm n_z^\pm >0$ so that the bulk modes are described by two *elliptic* equations [cf. Eq. (5)]. This condition is crucial to form the photonic Weyl system in the chiral metamaterial, which will be discussed later (cf. Results: Photonic Weyl system.). As a result, the bulk modes are represented by two concentric ellipsoids in the wave vector space. Note that the bulk modes for opposite sign of the chirality parameter are identical because of the symmetry about $\gamma$ [cf. Eq. (5)]. Here, the material parameters are arranged such that $n_t^+n_z^+>1$, $\left( n_t^+\right) ^2>1$ and $n_t^-n_z^-<1$, $\left( n_t^-\right) ^2<1$. The bulk modes are therefore either completely inside or completely outside the vacuum dispersion spheroid: $k_x^2+k_y^2+k_z^2=k_0^2$, as shown in Fig. 1(b) for the bulk modes on the half space ($k_y>0$). Note also that the bulk modes in Fig. 1a, b are represented by the same ellipsoids, although $n_s^->0$ ($s=t,z$) for the former and $n_s^-<0$ for the latter. The wave propagations in the two cases, however, are different in the issue of negative refraction and backward wave^64,65^. In the isotropic case, the inner bulk mode at the critical condition: $|\gamma |=\sqrt{\varepsilon \mu }$ (cf. Results: Bulk modes.) is reduced to a point at the origin.

Recall that the effective Hamiltonian in the present problem consists of two subsystems of the hybrid modes. Each subsystem is described by the spin-orbit Hamiltonian with spin 1 (cf. Results: Spin-orbit Hamiltonians.) and characterized by nonzero topological invariants (cf. Results: Topological invariants.). In this regard, the chiral metamaterial is considered a photonic analogue of the topological phase.

**Figure 2:**
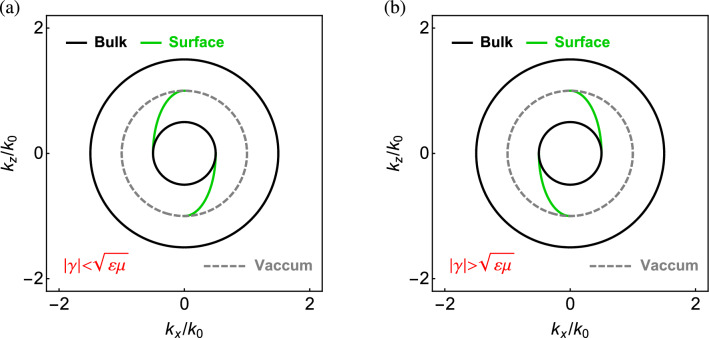
Surface modes at the interface between two chiral metamaterials with opposite sign of the chirality parameter and (**a**) $\varepsilon _n=\mu _n=1$ and $\gamma =\pm 0.5$ (**b**) $\varepsilon _n=\mu _n=0.5$ and $\gamma =\pm 1$ ($n=t,z$).

### Surface modes

Figure 2 shows the surface modes at the interface between vacuum ($y>0$) and the chiral metamaterial ($y<0$) in the $k_x$–$k_z$ plane based on Eq. (17). The bulk modes at $k_y=0$ are overlaid in the same plots. For clarity, we discuss the surface modes in the isotropic case, where $\varepsilon _n=\varepsilon$ and $\mu _n=\mu$ ($n=t,z$), and the analytical formulation for the surface modes are available. In particular, the surface modes are represented by a pair of curve segments symmetric about the center, which are located in the second and fourth quadrants for $\gamma <\sqrt{\varepsilon \mu }$ [cf. Fig. 2a], and the first and third quadrants for $\gamma >\sqrt{\varepsilon \mu }$ [cf. Fig. 2b]. Note that the surface modes and bulk modes ’merge’ at the points: $\left( k_x,{k_z}\right) =\left( \pm |\sqrt{\varepsilon \mu }-\gamma | k_0,0 \right)$ for the chiral metamaterial and $\left( 0,\pm k_0\right)$ for vacuum.

The bulk modes for opposite sign of the chirality parameter are identical because of the symmetry about $\gamma$ [cf. Eq. (4) or (5)]. The surface modes are located in the common gap of bulk modes in the wave vector space and *tangent* to the bulk modes^18,22^, including the chiral metamaterial (black solid contour) and vacuum dispersion circle: $k_x^2+k_z^2=k_0^2$ (gray dashed contour). This feature follows from the fact that the surface modes must convert seamlessly into the bulk modes as they approach their termination points^66^. The evanescent depth of the surface mode grows until at the point where the surface mode merges with the bulk mode^22^. The bulk modes on the vacuum side are topologically trivial, while on the chiral medium side they are topologically nontrivial with nonzero topological invariants (cf. Results: Topological invariants.). The surface modes correspond to the topological phase transition between two distinct topological phases in the momentum space^53,67^, their existence being guaranteed by the bulk-edge correspondence. In particular, the Hamiltonian of the photonic system respects the pseudo TR symmetry (cf. Results: Pseudo time-reversal symmetry.), leading to the topological protection of photonic edge or surface states.

**Figure 3:**
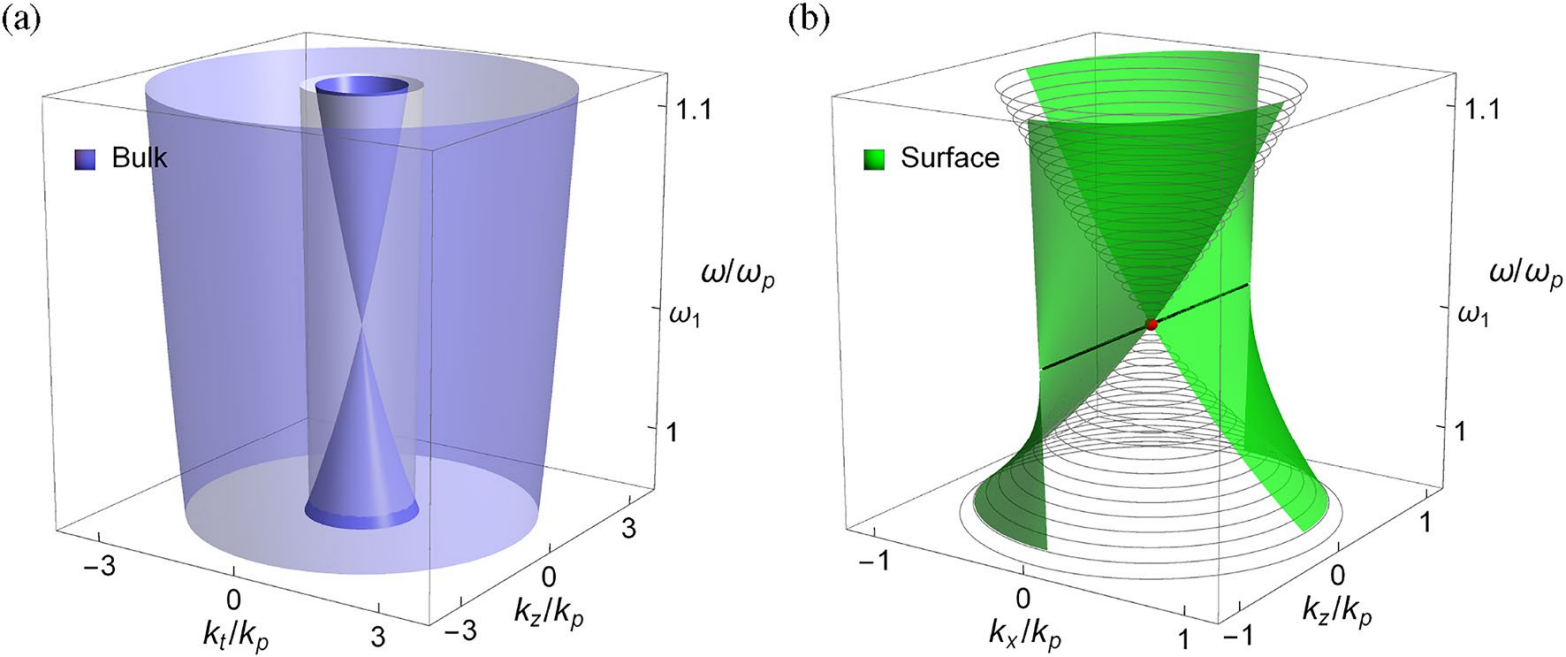
(**a**) Bulk modes and (**b**) surface modes in the frequency-wave vector space for the chiral metamaterial with $\varepsilon _{\infty }=4$, $\mu _{\infty }=3$, $\Omega _\mu =0.522$, $\Omega _\gamma =0.723$, and $\omega _0/\omega _p=0.8$. Wave vector components are scaled by $k_p=\omega _p/c$. In (a), white transparent cylinder is the dispersion surface of vacuum. In (b), bulk modes at constant frequencies for $k_y=0$ are outlined in gray mesh. Red dot is the Weyl point. Black line is the Fermi arc.

### Photonic Weyl system

Let the frequency dependence of the chiral medium be characterized by the Lorentz dispersion models: $\varepsilon = \varepsilon _\infty - \omega _p^2/\left( \omega ^2 - \omega _0^2 \right)$ and $\mu = \mu _\infty - \Omega _\mu \omega ^2/\left( \omega ^2-\omega _0^2\right)$, which are usually employed in the study of metamaterials^68^. Here, $\omega _p$ is the plasma frequency and $\omega _0$ is the resonance frequency. The chirality parameter is given by $\gamma = \Omega _\gamma \omega \omega _{p}/\left( \omega ^2 - \omega _0^2 \right)$, where $\Omega _\gamma ^2=\Omega _\mu$^69,70^. This model guarantees that the energy density in the underlying medium is positive definite (see Methods D).

Figure 3a shows the dispersion of bulk modes for the chiral metamaterial in the frequency-wave vector space. The bulk modes consist of a conic surface at the center of a cylindrical surface. In the present configuration, the material parameters are arranged such that $\varepsilon =\mu =\gamma =\frac{{{\varepsilon _\infty }{\mu _\infty }}}{{{\varepsilon _\infty } + {\mu _\infty }}}$ at the frequency $\omega _1= \sqrt{\omega _0^2 + \left( {{\varepsilon _\infty } + {\mu _\infty }} \right) \omega _p^2/\varepsilon _\infty ^2}$, where the inner bulk modes are reduced to a point at $\left( k_t,k_z,\omega \right) =(0,0,\omega _1)$. This is the condition that fulfills both the ’spin’-degenerate condition: $\varepsilon =\mu$ (cf. Results: Spin-orbit Hamiltonians.) and the critical condition: $|\gamma |=\sqrt{\varepsilon \mu }$ (cf. Results: Bulk modes.) in the present medium, which also forms the point-like degeneracy in the bulk modes. Here, $\omega _1$ is the transition frequency between the bulk modes with $n_->0$ and $n_-<0$ (cf. Results: Bulk modes.), and the bulk modes for either $\omega >\omega _1$ or $\omega <\omega _1$ are represented by similar elliptic curves. The former and the latter touch at a degenerate point, forming the conic surface for the inner bulk mode with $n_-$, while the outer bulk mode with $n_+$ (always positive) is a cylindrical surface. In this situation, the dispersion branch of the inner bulk modes resembles the linear crossing of valence and conduction bands in the *Weyl semimetal*^71^, with the crossing point known as the *Weyl point* and the associated frequency $\omega _1$ as the *Weyl frequency*. The topological charge associated with the Weyl point is consistent with the nonzero topological invariants of the system (cf. Results: Topological invariants.). Note that the inner bulk mode is reduced to a single point at the Weyl frequency. In this regard, the underling medium is considered a photonic analogue of the type-I Weyl semimetal^22^.

Note that in the absence of chirality ($\gamma =0$), the bulk modes are featured with the Dirac cone with fourfold degeneracy at the Dirac point: $(k_x,k_y,\omega )=(0,0,\omega _1)$ in the wave vector space [cf. Eq. (7)]. In the presence of chirality ($\gamma \ne 0$), the inversion symmetry is broken (cf. Results: Bulk modes.) and the fourfold degeneracy is lifted. As a result, the bulk modes are featured with a Weyl cone with twofold degeneracy at the Weyl point, along with a cylindrical surface [cf. Eq. (6)]. The topological charge carried by the Weyl point is consistent with the nonzero topological invariants $C_\pm =\pm 2$ of the present system (cf. Results: Topological invariants.). Here, the topological charge $\pm 2$ is associated with the *unconventional* spin-1 Weyl point with threefold linear degeneracy^46,72–74^. The net chirality vanishes in the Weyl semimetal, agreeing with the fact that the total Chern number is zero (cf. Results: Topological invariants.). Note also that there is only one Weyl point in the system, which is similar to the case of chiral metamaterial with $C_3$ rotational symmetry^46^. The feature of single Weyl point can be observed in the recent experimental study, where the Weyl point is surrounded by charged nodal walls^75^. There, however, may exist two^42,53^ or four^44,55^ Weyl points, depending on the constitutive relations of the metamaterials. A number of different Weyl points can be found in gyroid photonic crystals^40^ and non-symmorphic phononic crystals^74^. Very recently, higher-dimensional topological systems are proposed and expected to possess properties that their lower-dimensional counterparts do not support, such as Weyl surfaces and Weyl arcs^76,77^.

Figure 3b shows the dispersion of surface modes at the interface between vacuum and the chiral metamaterial in the frequency-wave vector space. For comparison, the bulk modes at constant frequencies for $k_y=0$ are outlined in gray mesh and overlaid in the same plot. Different from the surface modes in topological insulators that exist in the frequency (energy) band gap, the surface modes in gapless topological semimetals are defined in the region free of bulk modes at the same frequency (energy)^22^. Because of the frequency dependence of material parameters, the dispersion of surface modes is shown to be bended. In particular, the surface modes form a pair of spiral surface sheets wrapping around the Weyl cone. This feature is similar, though not identical, to the *helicoid* edge states that occur in the topological semimetals^23,44,72,78^. Similar features of helicoid-like edge states have also been observed in chiral metamaterials^46^.

Notice that the surface modes move to the other side of the $k_z$ axis as the frequency goes across the Weyl frequency $\omega _1$, where the dispersion of surface sheets experiences a smooth transition. At the Weyl frequency, the edge state that connects the Weyl point forms the so-called *Fermi arc*. The surface mode at the Weyl frequency is considered the Fermi arc-like edge state. In the present configuration, the two spiral surface sheets contact at the Weyl point and two associated Fermi arc-like edge states concatenate to yield a straight line segment.

In conclusion, we have investigated the photonic topological phases in chiral metamaterials characterized by the magnetoelectric tensors with diagonal chirality components. The underlying medium is considered a photonic analogue of the topological semimetal featured with a Weyl cone and a cylindrical surface in the frequency-wave vector space. Surface modes at the interface between vacuum and the chiral metamaterial exist in their common gap in the wave vector space, which are analytically formulated by algebraic equations. In particular, the surface modes form a pair of spiral surface sheets wrapping around the Weyl cone, resembling the helicoid surface states that occur in the topological semimetals.

## Methods

### Spin-orbit Hamiltonians

The wave equation for the hybrid modes $\mathbf{F^\pm }={\mathbf{{E}}\pm i \mathbf{{H'}}}$ in Eq. (3) can be rewritten as

18 $$\begin{aligned} {\tilde{\mathscr {H}_ \pm } }{{\tilde{\psi }} _ \pm } = {\tilde{\mathscr{D}}_ \pm }{\tilde{\psi _ \pm } }, \end{aligned}$$

where

19 $$\begin{aligned}{}&{\tilde{\mathscr {H}_ \pm } } = \pm c\left( {\begin{array}{*{20}{c}} {{k_z}} &{} {\frac{{{k_x} \mp i{k_y}}}{{\sqrt{2} }}} &{} 0 \\ {\frac{{{k_x} \pm i{k_y}}}{{\sqrt{2} }}} &{} 0 &{} {\frac{{{k_x} \mp i{k_y}}}{{\sqrt{2} }}} \\ 0 &{} {\frac{{{k_x} \pm i{k_y}}}{{\sqrt{2} }}} &{} { - {k_z}} \end{array} } \right) ,\end{aligned}$$

20 $$\begin{aligned}{}&{\tilde{\mathscr{D}}_ \pm } = \pm \omega \left( {\begin{array}{*{20}{c}} {{\varepsilon _t} \pm {\gamma _{t}}} &{} 0 &{} 0 \\ 0 &{} {{\varepsilon _z\pm \gamma _z}} &{} 0 \\ 0 &{} 0 &{} {{\varepsilon _t} \pm {\gamma _{t}}} \\ \end{array}} \right) , \end{aligned}$$

and $\tilde{\psi _ \pm } = {\left( - {\frac{{ {F_x^\pm } \mp i{F_y^\pm }}}{{\sqrt{2} }},{F_z},\frac{{{F_x^\pm } \pm i{F_y^\pm }}}{{\sqrt{2} }}} \right) ^T}$ are the *pseudospin* states that include $\pm \pi /2$ phase difference between the transverse hybrid field components (with respect to the optical axis of the medium)^57^. In the neighborhood of a *reference* frequency $\omega _\text {ref}$, ${\varepsilon _n}$ ($n=t,z$) can be approximated as ${\varepsilon _n} \approx {\varepsilon _{n0}} + {\left. {\frac{{d{\varepsilon _n}}}{{d\omega }}} \right| _{\omega = {\omega _\text {ref}}}}\left( {\omega - {\omega _\text {ref}}} \right) \equiv {\varepsilon _{n0}} + {\tilde{\varepsilon }_n}\delta \omega /{\omega _\text {ref}}$, where ${\tilde{\varepsilon }}_n$ is positive definite^57^. Taking into account of the frequency dispersion of the medium near the reference frequency, Eq. (18) is rearranged as a pair of eigensystems:

21 $$\begin{aligned} {\mathscr {H}'_ \pm }{\psi _ \pm } - {\mathscr{D}'_\pm }{\psi _ \pm } = \pm \delta \omega {\psi _ \pm }, \end{aligned}$$

where

22 $$ \begin{aligned}&{\mathscr{{H}}_ \pm ' } = \pm \frac{c}{{\sqrt{{{\tilde{\varepsilon }}_t}{{\tilde{\varepsilon }}_z}} }}\left( {\begin{array}{*{20}{c}} {\sqrt{\frac{{{{\tilde{\varepsilon }}_z}}}{{{{\tilde{\varepsilon }}_t}}}} }{k_z} &{} {\frac{{ {k_x} \mp i{k_y}}}{{\sqrt{2} }}} &{} 0 \\ {\frac{{ {k_x} \pm i{k_y}}}{{\sqrt{2} }}} &{} 0 &{} {\frac{{ {k_x} \mp i{k_y}}}{{\sqrt{2} }}} \\ 0 &{} {\frac{{ {k_x} \pm i{k_y}}}{{\sqrt{2} }}} &{} - {\sqrt{\frac{{{{\tilde{\varepsilon }}_z}}}{{{{\tilde{\varepsilon }}_t}}}} }{k_z} \\ \end{array} } \right) ,\end{aligned} $$

23 $$\begin{aligned}{}&{\mathscr{D}'_\pm } = \pm {\omega _{\mathrm{{ref}}}}\left( {\begin{array}{*{20}{c}} {\frac{{{\varepsilon _{t0}}}\pm \gamma _t}{{{{\tilde{\varepsilon}}_t}}}} &{} 0 &{} 0 \\ 0 &{} {\frac{{{\varepsilon _{z0}}}\pm \gamma _z}{{\tilde{\varepsilon }_z}}} &{} 0 \\ 0 &{} 0 &{} {\frac{{{\varepsilon _{t0}}}}{{{{\tilde{\varepsilon}}_t}}}\pm \gamma _t} \\ \end{array}} \right) , \end{aligned}$$

and ${\psi _ \pm } = {U^{ - 1}}{\tilde{\psi }_ \pm }$ with $U = \mathrm{{diag}}\left( {\sqrt{{{\tilde{\varepsilon}}_z}/{{\tilde{\varepsilon}}_t}} ,1,\sqrt{{{\tilde{\varepsilon}}_z}/{{\tilde{\varepsilon}}_t}} } \right)$. In the isotropic case, where ${\varepsilon _{t0}} = {\varepsilon _{z0}} \equiv \varepsilon$ and ${\tilde{\varepsilon }_t} = {\tilde{\varepsilon }_z} \equiv \tilde{\varepsilon }$, Eq. (21) is simplified to

24 $$\begin{aligned} {\mathscr {H}_ \pm }{\psi _ \pm } - \mathscr{D}_\pm {\psi _ \pm } = \pm \delta \omega {\psi _ \pm }, \end{aligned}$$

where

25 $$  \begin{aligned} \mathscr{{H}_ +} = v \mathbf{{k}} \cdot \mathbf{{S}},\quad \mathscr{{H}_ -} = - v \mathbf{{k}} \cdot {\mathbf{{S}}^*}, \end{aligned}  $$

and $\mathscr{D}_\pm = \pm {\omega _{\textrm{ref}}}\left( {\varepsilon \pm \gamma /\tilde{\varepsilon }} \right)$. Here, $v=c/{{\tilde{\varepsilon }}}$, $\mathbf{{k}}=k_x\hat{x}+k_y\hat{y}+k_z\hat{z}$, $\mathbf{{S}} = {S_x}\hat{x} + {S_y}\hat{y} + {S_z}\hat{z}$, with

26 $$\begin{aligned} {S_x} = \frac{1}{{\sqrt{2} }}\left( {\begin{array}{*{20}{c}} 0 &{} 1 &{} 0 \\ 1 &{} 0 &{} 1 \\ 0 &{} 1 &{} 0 \\ \end{array}} \right) ,\quad {S_y} = \frac{1}{{\sqrt{2} }}\left( {\begin{array}{*{20}{c}} 0 &{} { - i} &{} 0 \\ i &{} 0 &{} { - i} \\ 0 &{} i &{} 0 \\ \end{array}} \right) ,\quad {S_z} = \left( {\begin{array}{*{20}{c}} 1 &{} 0 &{} 0 \\ 0 &{} 0 &{} 0 \\ 0 &{} 0 &{} { - 1} \\ \end{array}} \right) \end{aligned}$$

being the spin matrices of spin 1, and * denoting the complex conjugate.

### Topological invariants

In terms of the spherical coordinates, the Hamiltonian $\mathscr{H}_\pm$ [cf. Eq. (25)] is rewritten as

27 $$ \begin{aligned} {\mathscr{{H}}_ \pm } = \pm \frac{{v |\textbf{k}|}}{{\sqrt{2} }}\left( {\begin{array}{*{20}{c}} {\sqrt{2} \cos \theta } &{} {{e^{ \mp i\phi }}\sin \theta } &{} 0 \\ {{e^{ \pm i\phi }}\sin \theta } &{} 0 &{} {{e^{ \mp i\phi }}\sin \theta } \\ 0 &{} {{e^{ \pm i\phi }}\sin \theta } &{} { - \sqrt{2} \cos \theta } \\ \end{array}} \right) , \end{aligned} $$

where $k_x= |\textbf{k}|\sin \theta \cos \phi$, $k_y= |\textbf{k}|\sin \theta \sin \phi$, and ${k_z} = |\textbf{k}|\cos \theta$ have been used. Here, $\theta$ and $\phi$ are the polar and azimuthal angles, respectively, on the surface *S*: $k_x^2 + k_y^2 + k_z^2= |\textbf{k}|^2$. The eigensystem for the Hamiltonian $\mathscr{H}_\pm$:

28 $$\begin{aligned} {\mathscr {H}_ \pm }\psi _ \pm ^\sigma =\lambda _ \pm ^\sigma \psi _ \pm ^\sigma \end{aligned}$$

is solved to give the eigenvalues $\lambda _ \pm ^\sigma = \sigma v |\textbf{k}|$ ($\sigma =\pm 1, 0$) and the normalized eigenvectors as

29 $$\begin{aligned}{}&\psi _ \pm ^\sigma = \frac{1}{2}\left( {\begin{array}{*{20}{c}} {\pm \sigma {e^{ \mp 2i\phi }}\left( {\pm \sigma + \cos \theta } \right) } \\ {\pm \sigma \sqrt{2} {e^{ \mp i\phi }}\sin \theta } \\ { 1 \mp \sigma \cos \theta } \\ \end{array} } \right) \quad (\sigma =\pm 1),\end{aligned}$$

30 $$\begin{aligned}{}&\psi _ \pm ^\sigma = \left( {\begin{array}{*{20}{c}} { - {e^{ \mp 2i\phi }}\sin \theta } \\ {\sqrt{2} {e^{ \mp i\phi }}\cos \theta } \\ {\sin \theta } \\ \end{array} } \right) \quad (\sigma =0). \end{aligned}$$

Note here that the eigenvalue $\lambda _ \pm ^\sigma$ is related to $\delta \omega$ in Eq. (24) as $\lambda _ \pm ^\sigma = \mathscr{D}_\pm \pm \delta \omega$. Based on Eqs. (29) and (30), the Berry connections $\textbf{A}_\pm ^\sigma =-i\left\langle {{\psi _\pm ^ \sigma }} \right. \left| {\nabla {\psi _\pm ^ \sigma }} \right\rangle$ are obtained as

31 $$\begin{aligned}{}&\textbf{A}_\pm ^\sigma =\mp \frac{1}{r}\left( \cot \frac{\theta }{2}\right) ^{\pm \sigma }\hat{\phi }\quad (\sigma =\pm 1), \end{aligned}$$

32 $$\begin{aligned}{}&\textbf{A}_\pm ^\sigma =\mp \frac{1}{r}\csc \theta \hat{\phi }\quad (\sigma =0). \end{aligned}$$

The Berry curvatures $\textbf{F}_\sigma =\nabla \times \textbf{A}_\pm ^\sigma$ are then given by

33 $$\begin{aligned} \textbf{F}_\sigma =\sigma \frac{{\hat{r}}}{{{r^2}}}\quad (\sigma =\pm 1, 0). \end{aligned}$$

Integrating over the sphere *S*, the Chern numbers ${C_\sigma } = \frac{1}{2\pi }\int _S {\textbf{F}_\sigma \cdot d\mathbf{{s}}}$ are calculated to give

34 $$\begin{aligned} {C_\sigma } = 2\sigma \quad (\sigma =\pm 1, 0). \end{aligned}$$

### Surface wave equation

According to Maxwell’s equations, the eigenfields on either side of the interface ($y=0$) are given by the nontrivial solutions of $\textbf{E}$ and $\textbf{H}$ [cf. Eq. (3)] or the *null space* of $\mathscr{H}_m$ [cf. Eq. (14)]. On vacuum side (say, $y>0$), we have

35 $$\begin{aligned}{}&{\mathbf{{E}}^{(1)}} = \frac{1}{{k_0^2}}\left( { - {k_x}k_y^{(0)},k_x^2 + k_z^2, - k_y^{(0)}{k_z}} \right) ,\quad {\mathbf{{H}}^{(1)}} = \frac{1}{{{\eta _0}{k_0}}}\left( { - {k_z},0,{k_x}} \right) , \end{aligned}$$

36 $$\begin{aligned}{}&{\mathbf{{E}}^{(2)}} = \frac{1}{{k_0^2}}\left( {{k_x}{k_z},k_y^{(0)}{k_z},k_z^2 - k_0^2} \right) ,\quad {\mathbf{{H}}^{(2)}} = \frac{1}{{{\eta _0}{k_0}}}\left( { - k_y^{(0)},{k_x},0} \right) , \end{aligned}$$

where $k_y^{(0)} = \sqrt{k_0^2 - k_x^2-k_z^2}$ is the normal (to the interface) wave vector component in vacuum. On the chiral medium side ($y<0$), the eigenfields are given by

37 $$\begin{aligned}{}&{\mathbf{{E}}^{(3)}} = \frac{1}{{k_0^2}}\left( { - {n _ + }{k_0}k_y^{(1)} - i{k_x}{k_z},{n _ + }{k_0}{k_x} - ik_y^{(1)}{k_z},i\left( {n _ + ^2k_0^2 - k_z^2} \right) } \right) ,{\mathbf{{H}}^{(3)}} = - \frac{i}{{{\eta _0}}}\sqrt{\frac{\varepsilon }{\mu }} {\mathbf{{E}}^{(3)}}, \end{aligned}$$

38 $$\begin{aligned}{}&{\mathbf{{E}}^{(4)}} = \frac{1}{{k_0^2}} \left( { - {n _ - }{k_0}k_y^{(2)} + i{k_x}{k_z},{n _ - }{k_0}{k_x} + ik_y^{(2)}{k_z}, - i\left( {n _ - ^2k_0^2 - k_z^2} \right) } \right) ,{\mathbf{{H}}^{(4)}} = \frac{i}{{{\eta _0}}}\sqrt{\frac{\varepsilon }{\mu }} {\mathbf{{E}}^{(4)}}, \end{aligned}$$

where $${n _ \pm } = \sqrt{\varepsilon \mu } \pm \gamma $$, and $$k_y^{(1)} = - \sqrt{n _ + ^2k_0^2 - k_x^2 - k_z^2}$$, $$k_y^{(2)} = - \sqrt{n _ - ^2k_0^2 - k_x^2 - k_z^2}$$ are the normal wave vector components in the chiral medium. Note that the eigenfields in Eqs. (35)–(38) share the common tangential wave vector components $$k_x$$ and $$k_z$$ across the interface, as a direct consequence of the phase matching of electromagnetic fields. For the surface waves to exist on the vacuum side ($$y>0)$$, $$k_y^{(0)}$$ should be purely imaginary with a positive value, so that the waves decay exponentially away from the interface. On the chiral medium side ($$y<0$$), $$k_y^{(1)}$$ and $$k_y^{(2)}$$ should be purely imaginary with a negative value for a similar reason.

The tangential electric and magnetic field components are continuous at the interface:

39 $$\begin{aligned}{}&C_1H_n^{(1)} + C_2H_n^{(2)} = C_3H_n^{(3)} + C_4H_n^{(4)}, \end{aligned}$$

40 $$\begin{aligned}{}&C_1E_n^{(1)} + C_2E_n^{(2)} = C_3E_n^{(3)} + C_4E_n^{(4)}, \end{aligned}$$

where $n=x,z$ and $C_1$, $C_2$, $C_3$, $C_4$ are constants. The existence of a nontrivial solution of these constants requires that the determinant of the 4 $\times$ 4 matrix obtained from Eqs. (39) and (40) be zero, which gives the characteristic equation of surface modes as

41 $$\begin{aligned} \begin{aligned}{}&\varepsilon \mu k_0^2\left[ {2\left( {{\gamma ^2} - \varepsilon \mu } \right) k_0^2 + 2\varepsilon \mu k_x^2 + \left( {\varepsilon + \mu } \right) {k_y^{(0)}}{k_y^{(1)}} + 2i\gamma {k_0}\left( {\left( {\varepsilon + \mu } \right) k_y^{(0)} - k_y^{(1)} - k_y^{(2)}} \right) } \right. \\&+ \left. {\left( {\left( {\varepsilon + \mu } \right) k_y^{(0)} - 2k_y^{(1)}} \right) k_y^{(2)}} \right] + 2i\sqrt{\varepsilon }\sqrt{\mu }\left( {\varepsilon \mu - 1} \right) \left( {k_y^{(1)} - k_y^{(2)}} \right) {k_x}{k_z}{k_0}\\&- \left[ {2\left( {{\gamma ^2} - 2} \right) \varepsilon \mu k_0^2 + 2k_x^2 - 2\varepsilon \mu k_y^{(1)}k_y^{(2)} + \left( {\varepsilon + \mu } \right) k_y^{(0)}\left( {k_y^{(1)} + k_y^{(2)}} \right) } \right. \\&+ \left. {2i\gamma {k_0}\left( {\left( {\varepsilon + \mu } \right) k_y^{(0)} - \varepsilon \mu \left( {k_y^{(1)} + k_y^{(2)}} \right) } \right) } \right] k_z^2 - 2k_z^4= 0. \end{aligned} \end{aligned}$$

### Electromagnetic energy density

The time-averaged energy density in a lossless medium is given by^79^

42 $$\begin{aligned} \left\langle W \right\rangle =\frac{ 1}{4}{V^\dag }{M}V, \end{aligned}$$

where

43 $$\begin{aligned} {M} = \left( {\begin{array}{*{20}{c}} {\frac{{\partial (\omega {\varepsilon _t })}}{{\partial \omega }}} &{} 0 &{} 0 &{} {i\frac{{\partial (\omega {\gamma _t})}}{{\partial \omega }}} &{} 0 &{} 0 \\ 0 &{} {\frac{{\partial (\omega {\varepsilon _t })}}{{\partial \omega }}} &{} 0 &{} 0 &{} {i\frac{{\partial (\omega {\gamma _t})}}{{\partial \omega }}} &{} 0 \\ 0 &{} 0 &{} {\frac{{\partial (\omega {\varepsilon _z })}}{{\partial \omega }}} &{} 0 &{} 0 &{} {i\frac{{\partial (\omega {\gamma _z})}}{{\partial \omega }}} \\ {-i\frac{{\partial (\omega {\gamma _t})}}{{\partial \omega }}} &{} 0 &{} 0 &{} {\frac{{\partial (\omega {\mu _t })}}{{\partial \omega }}} &{} 0 &{} 0 \\ 0 &{} { - i\frac{{\partial (\omega {\gamma _t})}}{{\partial \omega }}} &{} 0 &{} 0 &{} {\frac{{\partial (\omega {\mu _t })}}{{\partial \omega }}} &{} 0 \\ 0 &{} 0 &{} { - i\frac{{\partial (\omega {\gamma _z})}}{{\partial \omega }}} &{} 0 &{} 0 &{} {\frac{{\partial (\omega {\mu _z })}}{{\partial \omega }}} \\ \end{array}} \right) \end{aligned}$$

and $V=\left( \varepsilon _0 E_x,\varepsilon _0 E_y,\varepsilon _0 E_z,\mu _0 H_x,\mu _0 H_y,\mu _0 H_z \right) ^T$, with ${V^\dag }$ being the Hermitian conjugate of *V*. The energy density must be positive definite, which implies that both the trace and determinant of *M* are positive:

44 $$\begin{aligned} \textrm{Tr}\left( {M}\right)>0,\quad \textrm{Det}\left( {M}\right) >0. \end{aligned}$$

Based on the Lorentz dispersion model for the present medium (cf. Results: Photonic Weyl system.), these quantities become

45 $$\begin{aligned} \begin{aligned}{}&\mathrm{{Tr}}\left( {{M}} \right) =\frac{1}{{{{\left( {{\omega ^2} - \omega _0^2} \right) }^2}}} \cdot \\&\left[ {\left( {2{\varepsilon _{t\infty }} + {\varepsilon _{z\infty }} + 2{\mu _{t\infty }} + {\mu _{z\infty }}} \right) {{\left( {{\omega ^2} - \omega _0^2} \right) }^2} + 3\left( {{\omega ^2} + \omega _0^2} \right) \omega _p^2 - \omega \left( {{\omega ^2} - 3\omega _0^2} \right) \left( {2{\Omega _{\mu t}} + {\Omega _{\mu z}}} \right) } \right] \end{aligned} \end{aligned}$$

and

46 $$\begin{aligned} \begin{aligned}{}&\mathrm{{Det}}\left( {{M}} \right) = \frac{1}{{{{\left( {{\omega ^2} - \omega _0^2} \right) }^{12}}}} \cdot \\&{\left[ {\left( {{\varepsilon _{t\infty }}{{\left( {{\omega ^2} - \omega _0^2} \right) }^2} + \left( {{\omega ^2} + \omega _0^2} \right) \omega _p^2} \right) \left( {{\mu _{t\infty }}{{\left( {{\omega ^2} - \omega _0^2} \right) }^2} - {\omega ^2}({\omega ^2} - 3\omega _0^2){\Omega _{\mu t}}} \right) - 4{\omega ^2}\omega _0^4\omega _p^2\Omega _{\gamma t}^2} \right] ^2} \cdot \\&\left[ {\left( {{\varepsilon _{z\infty }}{{\left( {{\omega ^2} - \omega _0^2} \right) }^2} + \left( {{\omega ^2} + \omega _0^2} \right) \omega _p^2} \right) \left( {{\mu _{z\infty }}{{\left( {{\omega ^2} - \omega _0^2} \right) }^2} - {\omega ^2}({\omega ^2} - 3\omega _0^2){\Omega _{\mu z}}} \right) - 4{\omega ^2}\omega _0^4\omega _p^2\Omega _{\gamma z}^2} \right] , \end{aligned} \end{aligned}$$

both of which are positive in the present study.

## Data Availability

All data generated or analysed during this study are included in this published article.
